# High-Content, High-Throughput Screening for the Identification of Cytotoxic Compounds Based on Cell Morphology and Cell Proliferation Markers

**DOI:** 10.1371/journal.pone.0088338

**Published:** 2014-02-05

**Authors:** Heather L. Martin, Matthew Adams, Julie Higgins, Jacquelyn Bond, Ewan E. Morrison, Sandra M. Bell, Stuart Warriner, Adam Nelson, Darren C. Tomlinson

**Affiliations:** 1 BioScreening Technology Group, Leeds Institutes of Molecular Medicine, University of Leeds, Leeds, United Kingdom; 2 School of Chemistry, University of Leeds, Leeds, United Kingdom; 3 Section of Ophthalmology and Neuroscience, Leeds Institutes of Molecular Medicine, University of Leeds, Leeds, United Kingdom; 4 Astbury Centre for Structural Molecular Biology, University of Leeds, Leeds, United Kingdom; Biological Research Centre of the Hungarian Academy of Sciences, Hungary

## Abstract

Toxicity is a major cause of failure in drug discovery and development, and whilst robust toxicological testing occurs, efficiency could be improved if compounds with cytotoxic characteristics were identified during primary compound screening. The use of high-content imaging in primary screening is becoming more widespread, and by utilising phenotypic approaches it should be possible to incorporate cytotoxicity counter-screens into primary screens. Here we present a novel phenotypic assay that can be used as a counter-screen to identify compounds with adverse cellular effects. This assay has been developed using U2OS cells, the PerkinElmer Operetta high-content/high-throughput imaging system and Columbus image analysis software. In Columbus, algorithms were devised to identify changes in nuclear morphology, cell shape and proliferation using DAPI, TOTO-3 and phosphohistone H3 staining, respectively. The algorithms were developed and tested on cells treated with doxorubicin, taxol and nocodazole. The assay was then used to screen a novel, chemical library, rich in natural product-like molecules of over 300 compounds, 13.6% of which were identified as having adverse cellular effects. This assay provides a relatively cheap and rapid approach for identifying compounds with adverse cellular effects during screening assays, potentially reducing compound rejection due to toxicity in subsequent *in vitro* and *in vivo* assays.

## Introduction

Drug discovery and development is a multi-billion dollar industry in which the cost of failure for potential new drugs increases with their progression towards the clinic [1]. In this process, primary screening identifies potential lead compounds from large libraries of chemical compounds, the majority of which subsequent fail because of adverse effects – predominantly toxicity. Whilst the costs of primary screening have reduced in the last two decades as automation and high-throughput technologies advance, toxicity testing is still an expensive process despite of the use of *in vitro* cytotoxicity assays prior to *in vivo* testing [2]. Cytotoxicity is not the only adverse effect that causes compound failure as poor biopharmaceutical properties such as solubility and stability also contribute [1], but cytotoxicity is more difficult to predict. If identification of compounds with potentially adverse cellular effects could be combined with lead identification in a single assay this could reduce the subsequent drug failure rate and possibly the cost of drug discovery [3]. With the development of high-content, high-throughput imaging platforms with the ability to measure a variety of complex phenotypes, such integration is possible [4] and this technology has already been extended to explore the identification of known hepatotoxic compounds with the aim of improving in vitro identification of hepatoxins [3], [5]–[7]. The multiplex nature of these assays means they are a secondary line of investigation for potential lead compounds to eliminate those that induce liver toxicity. However, constitutive components of these assays may be useful for identifying compounds with sub-lethal adverse cellular effects or cytotoxic tendencies during primary screening fewer of these undesirable compounds are taken forward, thus potentially reducing compound attrition and the costs associated with this.

High-content/high-throughput imaging is based on the phenotypic assessment of a variety of biological activities. It requires clearly defined outputs into which individual cells may be assigned. However, the majority of published high-content screens use only two/three of the four channels available on the majority of these imaging platforms [4], [8]. One of these is normally a nuclear stain such as DAPI, Hoechst 33342 or DRAQ-5 that can be utilised to examine cytotoxicity by measuring loss of cells [4], [5]. Consequently one or more imaging channels are available to assess the potential of compounds to cause undesired side-effects on the target organ, particularly sub-lethal toxicity, concurrently with lead compound identification. Such assays may also be used in screens aiming to identify compounds from chemical libraries with the propensity to cause toxicity.

Herein, we show the development of a novel image analysis assay that identifies compounds with that have adverse cellular effects, often in conjunction with cytotoxic tendencies, using a high-content/high-throughput imaging approach. This technique has primarily been developed to be used us as an adjunct to targeted high-content, high-throughput primary screens to aid in the reduction of compound attrition due to adverse effects that become evident in subsequent testing.

## Methods

Human U2OS osteosarcoma cells (ATTC, VA) were maintained in Dulbecco's Modified Eagle's Medium (DMEM; PAA Laboratories GmbH, Pasching, Austria) supplemented with 10% foetal bovine serum (FBS; PAA) and 100 U/mL penicillin-streptomycin (PAA) at 37°C and 5% CO_2_. For screening, U2OS cells were plated into assay plates (96 well Viewpoint plates, Perkin Elmer, MA) at a density of 4000 cells/well in DMEM containing 10% FBS using an xrd-384 Fluid X dispenser fitted with an 8 nozzle resin dispensing cassette at 300 rpm (Fluid X Ltd, Nether Alderley, UK). Plates were allowed to equilibrate at room temperature in a hood for 1 hr then incubated for 24 hrs at 37°C and 5% CO_2_ before compound addition. Assays were developed and validated using taxol (resuspended in DMSO to 10 mg/mL; SigmaAldrich, Poole, UK), doxorubicin hydrochloride (10 mg/mL in DMSO; SigmaAldrich) and nocodazole (2 mg/mL in DMSO; SigmaAldrich) at a variety of concentrations.

### The Chemical Library

The screened library consisted of 329 skeletally-diverse compounds not previously used in cell-based assays, arrayed as 40 compound sets in duplicate in 96 well plates at a concentration of 10 mM in DMSO. Many of the compounds were natural product-like, and were based on highly diverse scaffolds reminiscent of either polyketide or alkaloid natural products. The synthesis of many of these compounds has been previously described [9]–[11], including an approach to natural product-like molecules of unprecedented scaffold diversity [11]. The library was diluted 1∶100 with DMEM containing 10% FBS on the day of compound addition and added to the plated U2OS cells at final concentration of 20 µM and a final DMSO percentage of 0.2%. Library addition was performed using a Bravo SRT liquid handling platform (Agilent Technologies, Wokingham, UK). Plates were assayed 24 and 48 hrs after compound addition using immunofluorescent staining and high-content imaging approaches as detailed below. Compound precipitation was visually assessed on addition and at imaging.

### Immunofluorescent Staining and Imaging

Immunofluorescent staining of assay plates was carried out as follows. Media was discarded and cells rinsed in phosphate buffered saline (PBS), before fixation in 4% paraformaldehyde (SigmaAldrich) for 15 mins. Cells were permeabilized with 0.1% Triton X-100 (VWR, Lutterworth, UK) in PBS for 5 mins, cells were then rinsed in PBS and blocked in 1% milk (Marvel, Premier Foods, St Albans, UK) for 5 mins before the addition of mouse anti-Histone H3 (phospho S10, 1∶4000; Abcam ab14955; Cambridge, UK) diluted in 1% milk for 1 hr at room temperature. Following PBS rinses, cells were incubated at room temperature for an hour in the dark with 1% milk containing goat anti-mouse AlexaFluor 568 (1∶1000; Molecular Probes, Eugene, OR) 1 µg/mL DAPI (Molecular Probes) and 500 nM TOTO-3 iodide (Molecular Probes). Following a final set of PBS washes, plates were scanned and images collected with an Operetta HTS imaging system (PerkinElmer) at 20× magnification with 12 fields of view (510×675 µm)/well. Images were then analysed with Columbus 2.2 (PerkinElmer).

### Apoptosis and Necrosis Identification

U2OS cells were plated and compounds added as described above, plates were then assayed 48 hrs after compound addition using a FITC Annexin V Apoptosis detection kit (BD Biosciences, Oxford, UK). Briefly cells were rinsed in PBS before the addition of 40 µl 1× binding buffer containing 2 µg/mL Hoechst, 25 µg/mL Annexin V and 2.5 µg/mL propidium iodide and incubated in the dark for 15 mins. A further 160 µl 1× binding buffer was then added and plates imaged immediately with the Operetta HTS imaging system as described above.

### Statistical Analysis

During assay development statistical analysis (Student T-tests, Mann-Whitney U tests and Spermann Rank correlation) was carried out using GraphPad Prism 6.00 software (GraphPad Software, La Jolla, CA). Assay validation was assessed by Z′-factor calculated as follows where *s* is the positive control and *c* the negative controls:



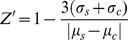



Z-scores were also calculated using the following formula:
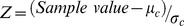
with hits determined as an absolute Z-score of 2 or more, as this correlates to a *P* value of 0.045, and thus statistical significance [12], [13]. False positives were defined as any DMSO-treated well in which the absolute Z score was greater than 2 whilst false negatives were taxol-treated wells which had an absolute Z score of less than 2 on three independent, randomly distributed test plates.

## Results and Discussion

### Assay Development

**Figure 1:**
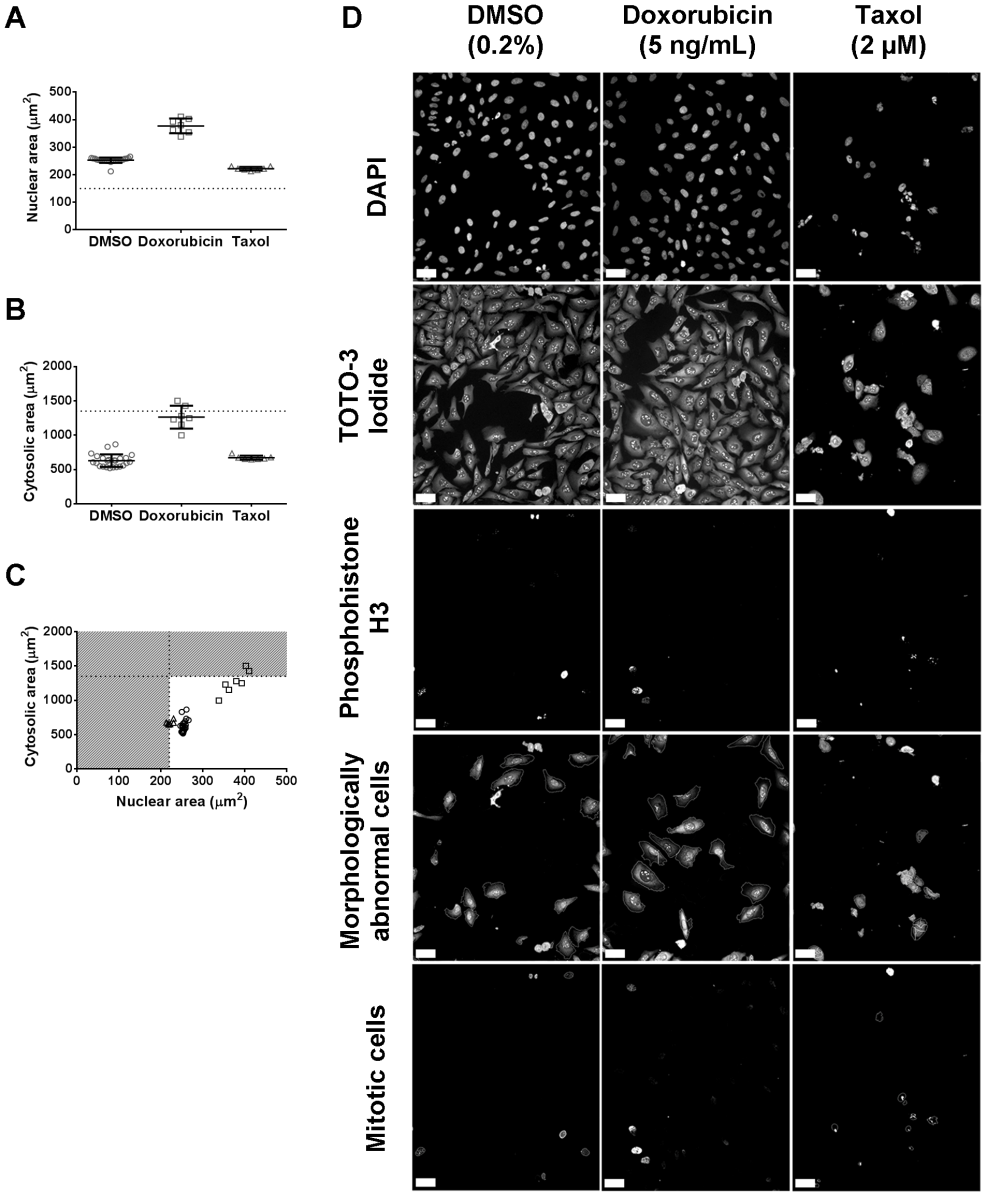
Development of image analysis protocols. Alterations in nuclear area A) and cytosolic area B) in cells treated with 5 ng/mL doxorubicin or 2 µM taxol compared with control (0.2% DMSO) were determined after 48 hours exposure. Dotted lines indicate the cut-offs used in the image analysis algorithm. C) Correlation between nuclear and cytosolic areas. A–C) Squares represent doxorubicin treated cells, circles 0.2% DMSO and triangles taxol treated cells, shading indicates the regions in which cells are deemed morphologically abnormal. D) Example images of the positive and negative controls showing the cells identified as morphologically abnormal or in mitosis. (Scale bars are 50 µm).

Phenotypic assessment of the adverse effects of compounds requires endpoints which may arise from the disruption of a variety of biological processes. For example, alterations to cell number can arise from the induction of apoptosis, necrosis, failure of cells to undergo mitosis, alterations to cell adhesion molecules or activation of oncogenic processes and increased cell division. Subsequently we sought to develop a novel image analysis algorithm to assess three phenotypic endpoints – cell number, the percentage of cells with morphological abnormalities and the percentage of cells in mitosis. As we aim to identify compounds with adverse cellular effects at an early stage of drug discovery we have chosen not to use cells from organs commonly affected by toxicity as these are not robustly used in high content/high throughput primary screens. Instead U2OS cells have been chosen for this initial study as they form well-spaced monolayers with a good nuclear to cytoplasmic ratio that are ideally suited to high-content, high-throughput imaging applications and are commonly used for primary screens [14]–[17]. However, the approach detailed below has the potential to be applied to a wide variety of cell types, including primary cells, as Lin et al [18] have shown only around 5% of toxic compounds demonstrate tissue specific cytotoxicity in *in vitro* assays. Thus the cell type would be selected based on the target molecule/phenotype for the compound set being tested. For example, for a compound set targeting a specific kinase, the assay would permit a comparison to be made between multiple cell lines, whose dependency on that kinase is known, thus enabling off-target cytotoxic effects to be assessed and the best lead compounds to be selected from the set. The first component of the algorithm was designed to assess alterations in cell number and morphology, for which it was necessary to define the normal cell morphology of U2OS cells. To induce morphological abnormalities and a reduction in cell number, cells were treated with taxol (2 and 0.1 µM) [19], doxorubicin (5 ng/mL) [20] or 0.2% DMSO (as a negative/vehicle control) for 48 hours. In agreement with Eom *et al.* [20], low dose doxorubicin increased cell size (Figure 1 A–B), whilst both high and low dose taxol treatment induced cell condensation and reduced cell number to differing degrees [19]. Taxol and doxorubicin were selected as potential positive controls, as whilst not being true cytotoxicants, they are well-documented as having multiple cellular effects including inhibition of cell cycle, induction of apoptosis and stabilisation of microtubules, as well as being natural products derivatives [19]–[22]. Indeed taxol (paclitaxel) was detected as having adverse effects in the majority parameters assessed in the studies of both O'Brien and Persson, including affecting mitochondria membrane potential [5], [7]. Thus by not using pure cytotoxicants we may enhance the algorithm's potential to detect subtle and pre-lethal changes in cell morphology. Using these alterations an image analysis algorithm was created using pre-designed building blocks within the Columbus software. Cell recognition was based on identification of nuclei as stained with DAPI, and cytosolic extent was based on TOTO-3 iodide staining, which was used at a sufficient concentration to permit staining of both DNA and RNA [23] and provided accurate identification of cell edge and discrimination between adjacent cells in fixed cells where permeabilization has compromised membrane integrity [24], and border objects were excluded. This nuclear identification was used as a measure of cell number, before extension of the algorithm to distinguish between doxorubicin treated cells and those treated with DMSO based on either increased cytosolic or nuclear area as determined by the staining extent of TOTO-3-iodide and DAPI respectively (Mann Whitney U test p<0.0001 for both cytosolic and nuclear area; Figure 1 A–B). Cytosolic and nuclear areas correlated (Spearman correlation p = 0.0035; Figure 1 C), meaning only one parameter needed to be used to identify enlarged cells. Cells with a nuclear area of less than 150 µm^2^ were also deemed to be abnormal. The identification of cytotoxic compounds based on cell morphology has been previously explored as a combined alterative to the classical battery of *in vitro* cytotoxicity tests [5], [7]. In these studies a strong marker for cytotoxicity was altered nuclear morphology, in particular nuclear size, which is in agreement with our findings [5], [7]. Both of these studies used combinatorial analysis of a variety of endpoints to identify known hepatotoxins, such an approach is not compatible with our aim of creating an adjunct assay to aid in data triage and hit selection. However further analysis parameters were required as taxol treated cells with altered morphology could not be easily distinguished from DMSO treated cells with a size cut-off alone and so it was necessary to add an additional parameter. As the nuclear area (and concurrently cytosolic area) of DMSO treated cells was similar to that of taxol treated cells (Figure 1 A), cells with a nuclear area of between 200 and 350 µm^2^ were then examined to see if differences in other nuclear morphology measures could be identified. No significant differences were identifiable (data not shown). However, even at the lower dose of taxol significant cell death occurred (Figure 1 D) (cell numbers were 54.6%±2.4 of DMSO; Student T-test p<0.0001). Visual examination of the images revealed that the abnormal cells that were not being picked up were those with an increased DAPI staining intensity. An intensity cut-off was therefore introduced at 1500 units to detect the small percentage of taxol treated cells with abnormal morphology not picked up previously (Figure 1). These parameters were then combined in a single image algorithm that identifies both alterations in cell number and the percentage of cells with abnormal morphology. Thus morphologically abnormal cells were defined as those cells with a cytoplasmic area of greater than 1350 µm^2^, a reduced nuclear area of less than 150 µm^2^ or with a mean DAPI intensity of greater than 1500 units. The reliability of these definitions was confirmed by visual inspection.

A second algorithm component was added to identify mitotic cells, defined as those cells with a maximum nuclear phosphohistone H3 intensity of greater than 1200 units (the maximum nuclear intensity for negative control cells was 225±1.8 units). This approach has been used previously to identify and characterize inhibitors of the Aurora kinases [25]. It gives information on compounds with sub-lethal effects including cytostatic effects, as compounds that induce alterations in cellular metabolism often induce changes to the number of cells undergoing mitosis and are likely to be detected in addition to those having a direct influence on mitosis [26]. Such compounds are commonly rejected during *in vitro* cytotoxicity tests, which assess ATP production and metabolic status amongst other endpoints, so their identification and removal at an earlier stage is beneficial in efficacy and financial terms [2].

### Assay Validation

**Figure 2:**
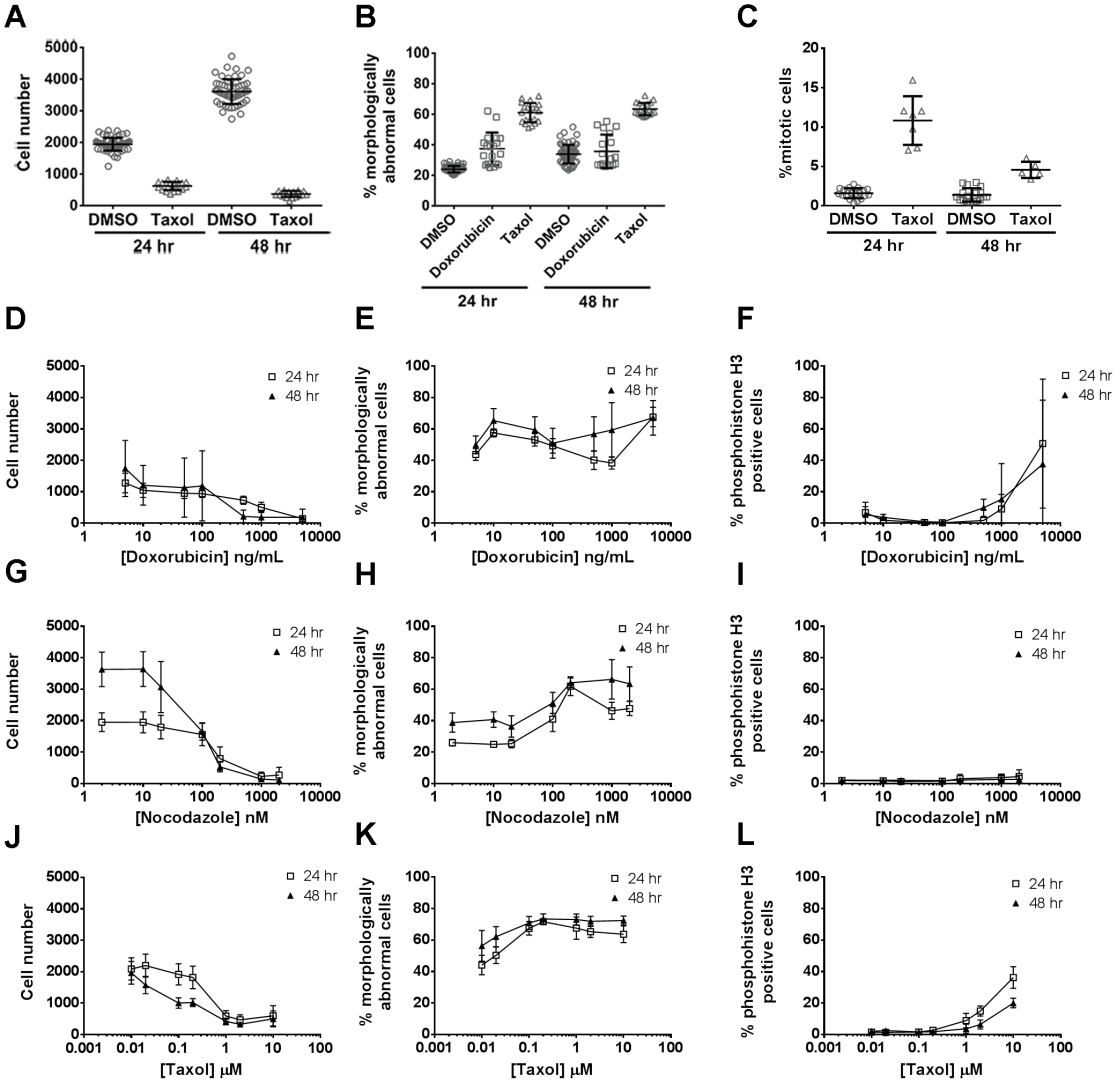
Validation of image analysis protocols. Clear delineation between positive (taxol and doxorubicin) and negative (0.2% DMSO) controls for the three endpoints assessed, cell number A), percentage morphologically abnormal cells B) and the percentage mitotic cells C) were observed after 24 or 48 hours exposure. Dose-response curves for doxorubicin (D–F), nocodazole (G–I) and taxol (J–L) for the three phenotypic endpoints assessed in this study. (Open squares are 24 hours after drug addition; closed triangles represent analysis 48 hours after drug addition).

The three endpoints in the image algorithm presented here can be split into individual algorithms based on the three channels used and added to a targeted primary screen as required, dependent on the number of free imaging channels. Z' factors were therefore individually calculated for the 3 phenotypic endpoints after both 24 and 48 hour exposures (Table 1) and were within acceptable ranges for cell number and percentage of morphologically abnormal cells [13], especially as moderate Z' factors can yield good quality data in a high-content setting [12]. However, whilst within range for the Z' factors were, in cases, lower than the ideal 0.5 cut-off especially for cell number at the 24 exposure. Low Z' factors arise from two main sources large variability within the dataset or a small separation window between the positive and negative controls. In the case of cell numbers after 24 hour treatment there is contribution from both sources as the coefficient of variance of the taxol treated wells is 21%, this is similar to that at 48 hours however at the latter timepoint the signal window is approximately 2.5 times larger giving the improved Z' factor. As low Z' factors influence our proposed hit detection method we calculated the Z scores for all the 2 µM taxol treated wells after 24 hours and all wells had a score of −6.5 or less indicating that the assay had good sensitivity as the variability was in the positive controls rather than the negative ones used in the Z score calculation. The Z' factor values for assessment of phosphohistone H3 levels were deemed to be sub-optimal for screening, as they were below 0 indicating signal overlap, [12], [13] this parameter was not evaluated as part of the test screen, but images from hit compounds were examined for increased numbers of mitotic cells. These data indicated that the two concentrations of taxol (0.1 and 2 µM) and 0.2% DMSO would be appropriate positive and negative controls respectively (Figure 2 A–C). The full algorithm was then further validated on cells treated with a variety of concentrations of nocodazole to confirm the algorithm could detect changes induced by compounds other than doxorubicin and taxol, and demonstrated dose-dependent responses at both 24 and 48 hour exposures for all three compounds (Figure 2 D–L). False positives and false negatives rates were calculated based on test plates dosed with 0.2% DMSO and taxol (0.1 and 2 µM) in an arbitrary layout (Table 1). The rate of false positive and false negatives predicted by analysis of the test plates is less than 5% for both cell number and the percentage of morphologically abnormal cells, indeed no false negatives were detected giving confidence that hits are unlikely to be missed.

**Table 1:** Z' factors and false positive and negative rates for the three phenotypic endpoints.

		% morphologically abnormal cells	Number of cells	% phosphohistone H3 positive cells
**24 hour exposure**				
	Z' Factor	0.55	0.32	−0.21
	False positive	0%	3.1%	0%
	False negative	0%	0%	0%
**48 hour exposure**				
	Z' Factor	0.40	0.57	−0.77
	False positive	3.3%	1.6%	0%
	False negative	0%	0%	13%

Z' factors, false positive and negative rates were calculated from test plates of U2OS cells treated with taxol (0.1 and 2 µM, n = 20 for each) or 0.2% (n = 62) DMSO in a random distribution.

To further ascertain if the changes seen in both cell number and morphology just represented cell death we assessed the percentage of cells in early apoptosis and the percentage of late stage apoptotic/dead necrotic cells using annexin V and propidium iodide staining in cells treated for 48 hours with 2 µM taxol or DMSO. The percentage of late stage/dead cells (2.90±1.04%) or early apoptotic cells (1.91±0.53%) in DMSO treated wells were low as anticipated. In contrast the percentage of late stage/dead cells (10.58±2.14%) or early apoptotic cells (13.2±1.77%) in taxol treated wells were significantly increased (P<0.001 and P<0.01 respectively). These data show that 20–30% of the cells detected as being morphologically abnormal are actually undergoing apoptosis or necrosis, suggesting that other cellular changes which are induced by compound treatment contribute to this phenotype, it also suggests that the reduction in cell numbers seen with taxol treatment are not solely the result of cytotoxicity which is reflected in the Z' factors being lower than could be anticipated with pure cytotoxicants. This correlates with the known mechanism of action of taxol as both an inducer of cell death as well as an inhibitor of cell proliferation [22], potentially enhancing the value of taxol as a positive control compared to pure cytotoxics. Thus this screening approach based alterations to both nuclear and cytosolic morphology appears to have the ability to detect adverse cellular changes that may not be reflected by gross cell death. Further studies are needed to determine which cell injury mechanisms, for example ATP depletion, mitochondria damage or generation of reactive oxygen species, are being reflected in the morphological changes seen.

### Test Screen

**Figure 3:**
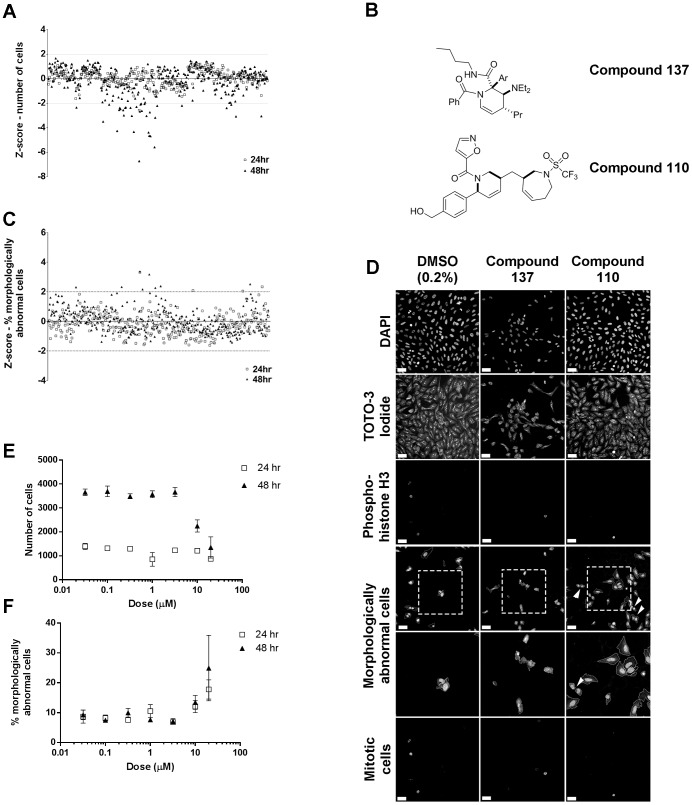
Screening a novel chemical library. A) Alterations in cell number A) after 24 (open squares) or 48 hours exposure (closed triangles) exposure to 329 diverse compounds, based on Z-scores calculated from negative controls, compounds above and below the cut-offs of +2 or −2 (dotted lines) are considered hits. B) Chemical structures of compounds 137,110 and 147 which were hit compounds in the three different endpoints assessed. C) Alterations in the percentage of morphologically abnormal cells after 24 (open squares) or 48 hours exposure (closed triangles) dotted lines as for A). D) Representative images from negative control (0.2% DMSO) and hit compounds in the three different endpoints assessed; compound 137 altered cell number and compound 110 increased the percentage of morphologically abnormal cells. (DMSO, compounds 137 and 110 after 48 hours exposure) (Scale bars are 50 µm). The most active compound, 137, showed dose-responses for both cell loss E) and increased the percentage of morphologically abnormal cells F).

The staining and image analysis protocols developed here were then applied to a library of 329 compounds, rich in natural product-like molecules, which have not been previously assayed. These compounds screened in quadruplicate at a final concentration of 20 µM with both 24 and 48 hour exposures. This is one fifth the concentration of previous studies [5], [7] as higher concentrations could not be used as U2OS cells were sensitive to DMSO at concentrations above the 0.2% used in these experiments. Eight positive and eight negative controls were used per screening plate. The main positive controls used were 100 nM and 2 µM taxol, with additional controls of 5 ng/mL doxorubicin and 100 nM nocodazole were also included. Z-scores were calculated for each of the two main phenotypic endpoints based on the negative controls (0.2% DMSO) for the screening batch (9 plates, 72 negative controls/batch and 18 of each positive control/batch (72 positive controls overall/batch)). As our screening concentrations were lower than previous studies and subtle effects more likely compounds were defined as hits if their absolute Z-score was greater than 2, a less stringent cut-off than the usual 3 [27] whilst retaining statistical significance (a Z score of 2 correlates to a p value of 0.045). By visual examination a single compound precipitated out and was subsequently excluded from further analysis. Thirty-four compounds which reduced cell number after 48 hours exposure (Table S1), but only one compound, number 137, that induced cell loss at both exposure times (Figure 3A–B). Compound 137 also increased the percentage of morphologically abnormal cells at both exposure times (Figure 3C). A correlation between altered cell number and morphological abnormality was also seen with compounds 151 and 160 after 48 hours exposure. Additionally five compounds, (42,110,141,168, and 301) increased the percentage of morphologically abnormal cells after 48 hours, but did not alter cell number (Figure 3C). Visual examination of images showed that compounds 110 and 168 showed multiple instances of two cells with small nuclei in close proximity, suggesting there may be alterations to daughter cells moving apart after cytokinesis or inhibited cell growth after cytokinesis (Figure 3D), such a phenotype would not be detected in cytotoxic assays and may have undesired side-effects if the compound was taken forward. The most active hit, compound 137, was then tested for dose-dependent responses which were evident at the top doses of 10 and 20 µM respectively (Figure 3 E–F). Thus, the algorithm created here has the capacity to detect compounds with adverse cellular effects as both outright cell loss or as a sub-lethal alteration to cell morphology/cell proliferation from a novel and structurally diverse chemical library with a hit rate of 10% and 3.6% respectively. This is the first report we are aware of that uses non-liver derived cell line to examine cytotoxicity by high content imaging in conjunction with a novel unenriched chemical library. So whilst the hit rates are lower than those reported by O'Brien and Persson [5], [7], the studies are not directly comparable as our library predominantly consists of compounds with natural product-like backbones and is not enriched for cytotoxicity compounds. In addition the maximal concentration and exposure time used in our study were also lower than the previous studies.

### Conclusion

In this study we sought to generate a novel high-content image analysis algorithm for use in primary screening assays to identify compounds with adverse cellular effects, by detecting abnormalities in cell morphology and mitotic delay/arrest which are indicators of likely cytotoxicity. The assay was initially optimised using agents known to have adverse cellular effects including cytotoxicity, showing a low false negative rate, and then tested using a previously untested chemical library rich in natural product-like molecules to validate the approach. Our study suggests that using alterations to cell morphology, particularly nuclear morphology, to identify adverse cellular effects during primary screening will be a valid approach to the triage of compounds to identify those likely to fail at later stages in the drug discovery pipeline. This is in concordance with the studies of O'Brien and Persson [5], [7] using known hepatotoxicants and HepG2 cells showing that nuclei morphology, specifically nuclear area, was a sensitive endpoint. However, additional testing of this endpoint with a wider selection of compounds for which the mechanism of action has been identified is required to further strengthen the validation of this assay. This would in conjunction with assessments of ATP status and mitochondrial damage help to determine which mechanisms of cellular injury this endpoint can detect. The algorithm components described here does not seek to provide detailed mechanistic data, or identify lead compounds (unless cytotoxicity is the desired endpoint), but they do have the power to provide valuable data on compound induced adverse effects as adjuncts in primary screens. This algorithm is designed for screens where adverse cellular effects are not the primary output as a number of high-content, high-throughput screens looking at detecting known hepatotoxins have already been published [3], [5], [7]. These studies utilise high content imaging to its full capacity showing the power of this technology for detecting adverse cellular effects. However, these approaches will not currently replace *in vitro* toxicity testing *in toto* as they have not yet been validated in cell types other than liver with compounds displaying toxicity that is not solely hepatotoxicity, admittedly the most difficult type of compound-induced toxicity to predict [28], and therefore a need for traditional toxicity testing still exists. It may only be a matter of time before such assays are routinely used for in vitro toxicity testing as considerable overlap occurs between the toxicity of hepatotoxic, nephrotoxic and cardiotoxic compounds in the cell lines commonly used to assess such adverse effects [18]. We feel that the current study further supports the use of a high-content approach to identifying cytotoxic compounds by use of a non-traditional cell-line in toxicity testing and a novel compound library. As the algorithms generated in this study are not as multiplexed as previous work [5], [7] they may be included in the design of primary screening assays to incorporate some degree of testing for adverse effects to potentially limit the number of compounds taken forwards from a primary screen that subsequently display adverse cellular effects, thereby minimising lead compound failure and reducing the costs associated with this. The assay presented here is an important step towards this aim, however, further work is now required to assess the performance of the assay in detecting adverse effects in a wider variety of cell lines treated with compounds with a greater range of toxic mechanisms, and in combination with targeted primary screens.

## Supporting Information

Table S1 **Identification of hit compounds from the test screen.** Structures of the screen library detected as have an absolute Z score greater than 2 for one or more of the phenotypes assessed; cell number, increased percentage of morphologically abnormal cells or the percentage of cells in mitosis.
(DOCX)
