# Supplementary material for: High-Content, High-Throughput Screening for the Identification of Cytotoxic Compounds Based on Cell Morphology and Cell Proliferation Markers
Source: PLoS One. 2014 Feb 5;9(2):e88338. doi: 10.1371/journal.pone.0088338 (PMC3914966; doi:10.1371/journal.pone.0088338)
Supplement: Table S1 — Identification of hit compounds from the test screen. Structures of the screen library detected as have an absolute Z score greater than 2 for one or more of the phenotypes assessed; cell number, increased percentage of morphologically abnormal cells or the percentage of cells in mitosis. (DOCX) [file pone.0088338.s001.docx]

| **Compound** | **Structure** | **Hit Phenotypes** |
| --- | --- | --- |
| 42 |  | Morphologically abnormal cells |
| 43 | 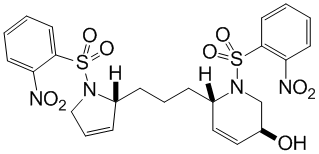 | Cell loss |
| 83 | 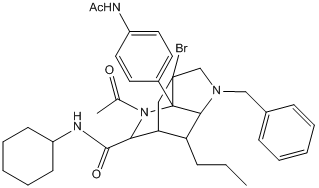 | Cell loss |
| 92 | 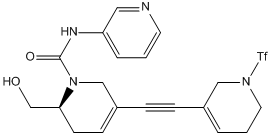 | Cell loss |
| 104 | 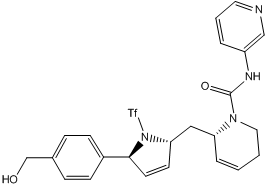 | Cell loss |
| 107 | 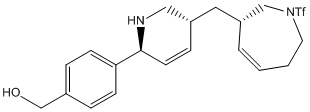 | Cell loss |
| 108 | 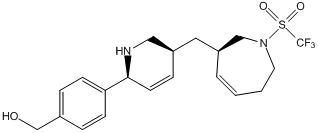 | Cell loss |
| 109 | 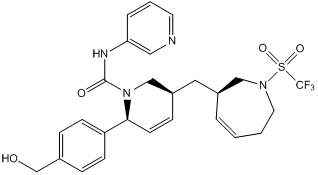 | Cell loss |
| 110 | 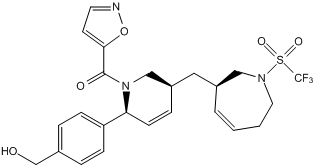 | Morphologically abnormal cells |
| 113 | 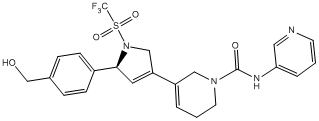 | Cell loss |
| 114 | 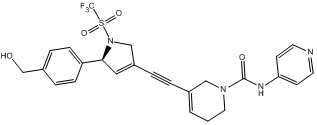 | Cell loss |
| 116 | 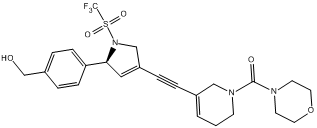 | Cell loss |
| 117 | 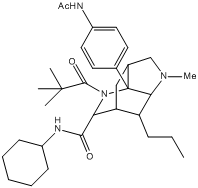 | Cell loss |
| 124 | 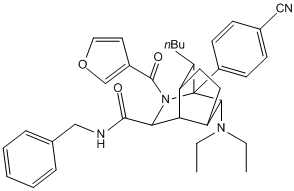 | Cell loss |
| 126 | 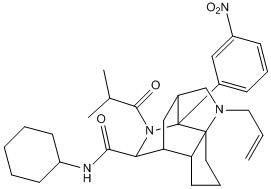 | Cell loss |
| 129 | 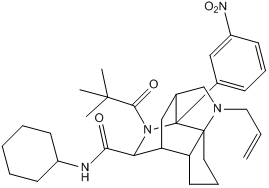 | Cell loss, morphologically abnormal cells |
| 133 | 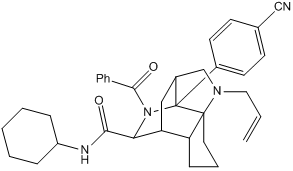 | Cell loss |
| 137 | 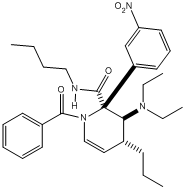 | Cell loss, morphologically abnormal cells |
| 138 | 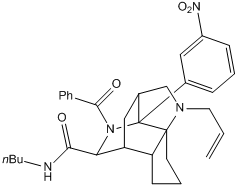 | Cell loss |
| 139 | 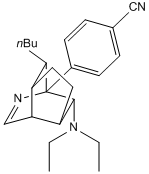 | Cell loss |
| 140 | 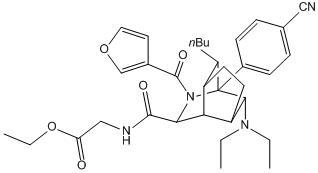 | Cell loss |
| 141 | 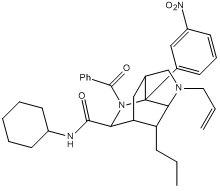 | Cell loss, morphologically abnormal cells |
| 145 | 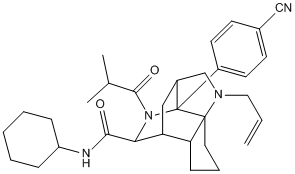 | Cell loss |
| 147 | 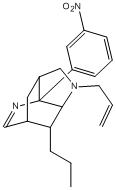 | Mitotic arrest/delay |
| 148 | 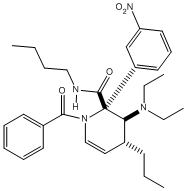 | Cell loss |
| 151 | 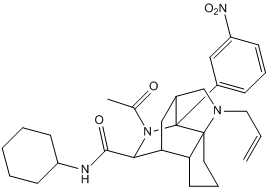 | Cell loss, morphologically abnormal cells |
| 152 | 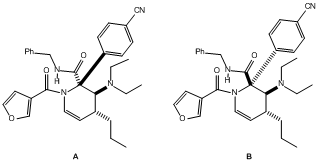 | Cell loss, morphologically abnormal cells |
| 153 | 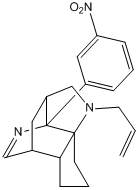 | Cell loss |
| 154 | 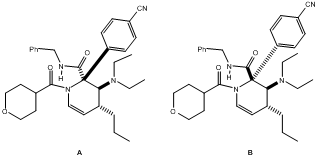 | Cell loss |
| 160 | 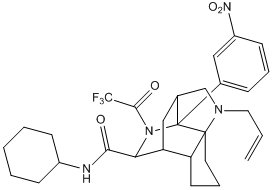 | Cell loss, morphologically abnormal cells |
| 163 | 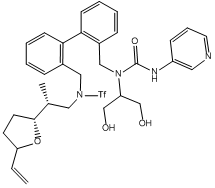 | Cell loss, morphologically abnormal cells |
| 164 | 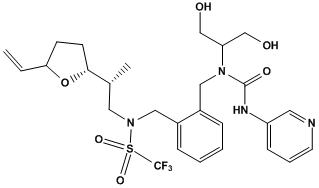 | Cell loss, morphologically abnormal cells |
| 168 | 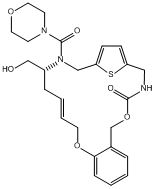 | Morphologically abnormal cells |
| 201 | 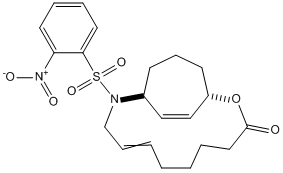 | Cell loss |
| 216 |  | Morphologically abnormal cells |
| 268 | 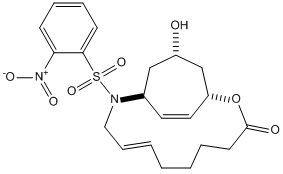 | Cell loss |
| 275 | 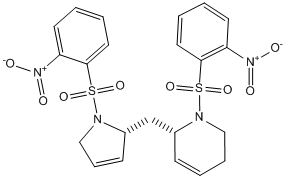 | Cell loss |
| 282 | 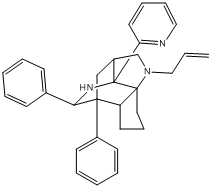 | Cell loss |
| 290 |  | Morphologically abnormal cells |
| 301 |  | Morphologically abnormal cells |
| 302 | 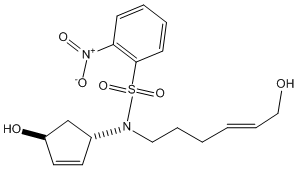 | Cell loss |
| 319 |  | Morphologically abnormal cells |
